# Virtual anthropology – a brief review of the literature and history of computed tomography

**DOI:** 10.1080/20961790.2017.1369621

**Published:** 2017-09-14

**Authors:** Tanya Uldin

**Affiliations:** Department of Medicine and Community Health, University Center of Legal Medicine Lausanne-Geneva, University Hospital of Lausanne, Lausanne, Switzerland

**Keywords:** Forensic science, forensic anthropology, virtual anthropology, palaeoanthropology, computed tomography, MDCT, bone imaging

## Abstract

Computed tomography (CT) has influenced numerous fields since its inception in the 1970s. The field of palaeoanthropology significantly benefited from this efficient and non-invasive medium in terms of the conservation, reconstruction and analysis of fossil human remains. Over the past decade, there has been a steady increase in the number of forensic anthropological studies incorporating virtual osteological analyses. Because of the increasing importance of these modern cross-sectional imaging techniques and the requirement for standardized parameters in forensic science, we deemed it important to outline the history and development of CT applications in these related academic areas. The present paper outlines the history of “virtual anthropology” and osteological multi-detector CT in the context of palaeoanthropology and forensic anthropology.

## Introduction

The use of digital imaging techniques, such as computed tomography (CT) or optical surface scanning, has contributed to numerous medical-related domains, including biology, palaeontology, biological anthropology, archaeology, forensic science and materials science. As a non-invasive diagnostic tool, these techniques have many advantages. The digitized object can be examined externally and internally, while being simultaneously manipulated without causing damage to the object. Investigations are repeatable and verifiable at any time, and digital data or 3D-printed hard copies of the object can be easily replicated and shared within the scientific community.

Over the past two decades, there has been a steady increase in the use of multi-detector computed tomography (MDCT), and the demand for specific data-acquisition and post-processing parameters in medical research has led to various recommendations and protocols [1–6]. However, different research areas require specific solutions. Indeed, the implementation of CT in palaeoanthropology and forensic anthropology arose to specifically address the needs of these fields. This review aims to outline the history of “virtual anthropology” and CT in the fields of palaeoanthropology and forensic anthropology.

## The use of CT in palaeoanthropology

The introduction of clinical CT by Hounsfield [7–9] led to immediate benefits for palaeoanthropology; the non-invasive aspect of the technique allowed researchers to conserve precious and often fragile fossils, prehistoric human skeletal remains and mummified remains [10,11]. Around the same time, specific scanning protocols and guidelines were developed for the technique [12–16]. Subsequent discussions by Tate and Cann [12] to modify the Hounsfield unit scale for the high density of fossil bones led to the development of an extended scale for the better visualization of internal structures. Sumner et al. [14] focused on improving the accuracy and precision of the measurements, providing a solution for the treatment of beam-hardening artefacts. In particular, the studies by Ruff and Leo [15] and Spoor et al. [16] highlighted the potential sources of error in this technique, such as inaccuracies in measurement due to partial volume effect and incorrect threshold values, or the problematic correlation of CT-number values and tissue densities depending on the X-ray beam energy (tube voltage); these issues still occur in modern medical research [17–19]. The authors also provided specific guidelines for bone scanning, image processing and interpretation of the data.

The development of spiral CT in 1989 [20] provided enhanced cross-sectional data acquisition and better image processing software for 3D surface reconstruction. This proved to be an advantage for the investigation of the Tyrolean Iceman, also known as “Ötzi”, a mummified corpse from the Chalcolithic period discovered at the Austrian–Italian border in the Alps in 1991 [21–24]. Full-body, 3-mm, spiral CT scanning was performed to examine the internal bony structures of the corpse. The high-resolution images obtained made it possible to detect particularly small fractures that would have remained invisible using conventional radiography. zur Nedden and colleagues [23,24] reconstructed the skull of the Tyrolean Iceman in 3D using post-processing software of the CT workstation and though 3D printing. They applied stereolithography, a computer-guided 3D printing technique that uses UV lasers and photohardening resin, to create a 3D model, layer by layer. The group then compared measurements from the real skull, the virtual skull and the model, and demonstrated the accuracy of the measurements. The study also shed light on various problematic artefacts, such as pseudo-lesions, which may occur due to volume averaging effects [23]; this problem has yet to be solved in medical research [25–27].

From as early as the 1990s, it became popular to digitize fossils in palaeoanthropological studies, no longer restricting data collection to CT alone. Optical surface scanning and micro-CT are two additional techniques commonly used according to the purpose of the research [28]. Weber and co-workers, who took part in the study of the Tyrolean Iceman, first coined the term “virtual anthropology” [29–31] as a multidisciplinary approach combining knowledge from related academic areas such as anthropology, palaeontology, primatology, medicine, mathematics, statistics, computer science and engineering [31]. According to the authors, the potential of digitized objects lies in the permanency of the data, and the accessibility to internal anatomical structures that would otherwise remain hidden during routine external examinations. Digitization also allows for reproducibility and the use of advanced analyses (i.e. geometric morphometrics), not to mention, the ease of data sharing [31]. CT-based research in palaeoanthropology using 3D reconstructions has focused primarily on morphometric and shape analyses to investigate human evolution [30,32–39]. To meet the complexity of this new field of research, two textbooks on virtual anthropology were published, providing profound technological insight and specific guidelines [28,40].

One of the most recent discoveries in palaeoanthropology – *Homo naledi*, an extinct hominin species from South Africa [41] – shows the positive effects of this “digital revolution”, and is a good example of the consistent application of Weber's [29] demand for “Glasnost in Palaeoanthropology”; i.e. free accessibility of digitized fossil data to enhance scientific progress and transparency. Weber and his co-workers published the first data-set of a hominid skull [29]. Fifteen years on, many scientists now publish their 3D data, i.e. surface scans of several skeletal parts of *Homo naledi* were made available (open-access) on the MorphoSource website, a data archive for 3D fossil data [42]. Making the data accessible allowed for the immediate exchange of information among the research community.

Palaeontology and zooarchaeology are two related academic areas of palaeoanthropology that have benefited from imaging techniques. MDCT and other digital visualization tools have been extensively used to reconstruct extinct species, apply advanced statistical methods and facilitate the sharing of data [43]. Additionally, comparative osteological collections have been established to highlight the anatomical variations in the skeleton among species. This is particularly advantageous when access to reference collections of real bones is restricted or unavailable [44,45]. du Plessis and co-workers [46] have demonstrated the potential of automated laser preparation, which is used to separate fossil bones and surrounding sediments. The technology is based on density differences in materials using data obtained from a micro-CT scanner. The authors found that preliminary planning and preparation in a virtual environment before the laser ablation of the rock prevents damage to the object and is also time-saving.

Current studies on craniometrics and virtual reconstruction [47,48] are showing a rising interaction among palaeoanthropology, medical research and forensic sciences. Benazzi and Senck [47] have explored different methods of virtual 3D reconstruction for preoperative planning incorporating knowledge and methods garnered from palaeoanthropology. Guyomarc'h et al. [48], in their comparison of different post-processing software, have discussed the uncertainties in measurements due to surface reconstruction, showing that every phase of digitization involves a certain risk of shape alteration. This problem affects not only palaeoanthropology or preoperative planning, but also facial reconstruction. These examples illustrate the importance of knowledge transfer from one discipline to another. Indeed, forensic medicine has benefited from advances in palaeoanthropology and medical research, but has also contributed to numerous methodological advances; this concept is further developed in the following section.

## Post-mortem MDCT and forensic anthropology

Radiographic techniques are well established in forensic medicine and forensic anthropology [49–51], and are used for trauma diagnosis and to identify unknown deceased remains by comparing individual features using ante- and post-mortem radiographs [52–54]. Quatrehomme et al. [55], for example, emphasized that trabecular bone morphology can lead to positive identification. Stephan and co-workers [56,57] developed a geometric–morphometric method of clavicle-shape comparison using optical surface scan and radiographs. Furthermore, Derrick et al. [58] have modified software used in spine injury diagnosis to identify vertebrae in ante- and post-mortem comparisons of radiographs. Plain radiography is inexpensive and easy to use, and methods specific to forensic radiography are still being developed. In contrast, the routine application of MDCT in forensic medicine and forensic anthropology is a relatively recent development, presumably because its high cost and limited accessibility previously hindered its regular use.

The term post-mortem computed tomography (PMCT) was introduced in the early 1980s by Krantz and Holtås [59], who used CT scanning to enhance autopsy findings in diving fatalities. However, CT was not used frequently until the mid-1990s. Reichs [60,61] was the first to compare radiographic and CT images of the frontal sinuses, and provided technical and methodological recommendations for standardization. Donchin et al. [62] conducted one of the first studies comparing whole-body CT scanning with the findings of conventional autopsy. The authors showed that while neither method was superior, combining the methods could potentially improve the results of medico-legal investigations. On the other hand, in studies evaluating the potential of CT data to enhance methods for forensic facial reconstructions, Phillips and Smuts [63] found that soft tissue thickness measurements obtained by CT were more accurate than those obtained by conventional methods. Using a semi-automated method, Quatrehomme et al. [64] presented various advantages and pitfalls of using CT data for 3D facial reconstruction.

About 10 years ago, Dirnhofer and co-workers [65,66] developed the “image-guided virtual autopsy” as a supporting tool for conventional autopsy techniques. As emphasized by Weber [29], there are several advantages of virtual autopsy, including the permanency of the digitized images, the reproducibility of the methods and the potential to share the data for more objective investigations.

At present, forensic imaging is routinely used in several medico-legal institutes and this has provided more opportunities for forensic anthropologists to use post-mortem MDCT. Hence, there has been a rapid surge in the use of post-mortem MDCT in forensic anthropology, with two main types of publications: (1) studies describing the generalized use of MDCT in disaster victim identification in the medico-legal context, and case reports, highlighting the utility of MDCT-imaging for specific cases [67–80]; (2) other studies have used MDCT to evaluate skeletal traits to build a database for the biological profiling of unidentified human remains. Most of these types of studies have used MDCT for age [81–100], sex [86,101–130], stature [116,119,128,131–137] and body mass [138–140] estimations; or to validate a range of general measurements [48,141–148]. Several other studies have compared conventional radiographic methods to MDCT: for example, MDCT has been used to compare ante- and post-mortem radiographic images of frontal sinus patterns [60,61,149] – which are reliable in positive identification [150,151] – and paranasal sinuses [152]. Other studies have tested the utility of MDCT to measure trabecular bone for estimations of age at death [81,84–86,96]. Wade et al. [90] and de Froidmont et al. [144] sought to compare conventional radiography and MDCT, both showing the superiority of MDCT over conventional radiography in the analysis of fine anatomical structures.

Improved access to MDCT devices in the past five years has led to an increase in the list of publications using this technique. By routinely using post-mortem MDCT, it is now possible to continuously collect digital data, which provides a foundation for sound research. Indeed, the work by Torimitsu et al. [122–126,133–136] and Zhang et al. [128-129,137] has resulted in scanning protocols that allow for better comparability and reproducibility.

Radiography and MDCT are also used to estimate age of the living. Specialists of different disciplines, including forensic pathologists, odontologists, radiologists and anthropologists take into consideration mainly physical, dental and osseous (hand wrist, medial clavicle) developmental changes to assess the age of minor or young adult individuals [153–155]. The methods used are mainly derived from paediatric radiology and odontology, and the acquisition protocols follow clinical guidelines to keep radiation doses as low as possible. MDCT acquisition parameters, such as tube potential, tube current, beam collimation, among others, must be adequately balanced to obtain appropriate image quality [2,156–159]. Schmeling and co-workers, who mainly explore the ossification of the medial epiphyses of the clavicle [158–161], have tested different reconstruction slice thicknesses to determine the optimal parameters for measurements. They recommend working with the thinnest slice thickness possible, as this parameter considerably influences the results of the ossification stages [162].

**Figure 1.**
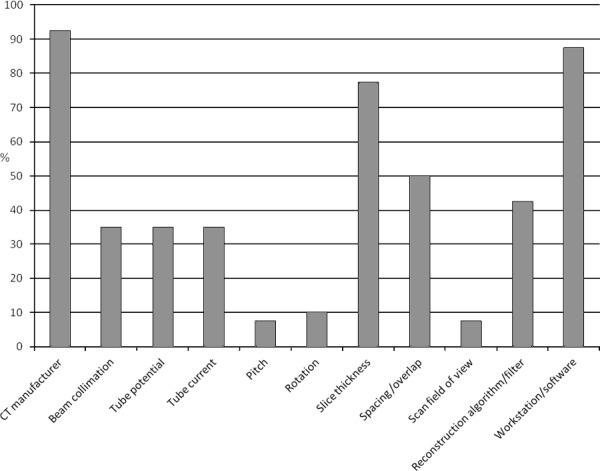
Quantity of acquisition parameters published in forensic anthropological studies from 2005 to 2015 (*n* = 40) [164, p. 22, Fig. 2].

Despite the increase in studies on MDCT, few have published appropriate technical parameters, which minimizes reproducibility and limits cross comparisons with other studies. In their review of the literature on CT examinations of human mummies, O'Brien et al. [163] criticized the lack of reproducibility due to insufficiently published technical parameters, indicating that clearly defined scanning protocols were missing in about one-third of papers (*n* = 31) published between 1979 and 2005. This is in line with our observations [164] during a review of forensic anthropological studies on MDCT bone imaging published between 2005 and 2015 (*n* = 40). While most studies mentioned the device manufacturer, post-processing software (or at least the workstation) and slice thickness, few (*n* = 8) published all parameters shown in Figure 1. Two current papers in mummy research have revisited this topic: Conlogue [165] described basic scanning parameters and discussed the advantages and limitations of MDCT applied to mummified human remains. Cox [166] criticized the lack of technical knowledge and standard parameters for MDCT. However, in forensic anthropology, there are an increasing number of papers that critically discuss the technical parameters of MDCT. Grabherr et al. [86] explain the influence of slice thickness and reconstruction filters and, as mentioned above, Guyomarc'h et al. [48] detail how surface reconstruction is affected by the choice of the segmentation algorithm. Likewise, Villa et al. [96] highlight the issues concerning the visualization of surface reconstruction of virtual bones, and emphasize that CT scanning parameters have an impact on surface reconstruction, as small osseous structures are improperly displayed.

The approaches to digitize bones differ between forensic anthropology and palaeoanthropology. The close connection between forensic anthropology and forensic medicine has meant that imaging techniques primarily used as diagnostic tools employ standard parameters taken from routine clinical assays. In addition, research has focused on the development and evaluation of methods that are used for identification.

## Conclusion and perspectives

Ideal research conditions for anthropologists would include a comprehensive collection of documented skeletons, with a balanced distribution of age and sex, and information pertaining to stature, weight and/or medical history. Routine post-mortem MDCT generates an invaluable data pool that could serve future research and method evaluation. The ease of accessibility, the permanency of the data and the non-invasiveness of the investigation has fuelled research using CT approaches in forensic anthropology over the past decade. However, until it becomes routine practice to publish the scan parameters, technical information and types of post-processing performed, the potential for MDCT will remain underused. Indeed, the choice of appropriate image processing software affects the data [48]. Comparative studies on post-processing parameters, such as segmentation algorithms, are thus also required so that adjustments can be made to standardize the data for its generalizability. Finally, there is a need to intensify the transfer of knowledge among palaeoanthropology, palaeontology, archaeology and other related academic fields. With almost 30 years of experience with bone imaging, research into forensic anthropology could profit from already-existing methods for better future solutions.
